# 50-year-old Male With Chest Pain

**DOI:** 10.5811/cpcem.2019.10.45318

**Published:** 2019-10-21

**Authors:** William L. Fernandez, Laura J. Bontempo, Zachary D.W. Dezman

**Affiliations:** * University of Maryland Medical Center, Department of Emergency Medicine, Baltimore, Maryland; † University of Maryland School of Medicine, Department of Emergency Medicine, Baltimore, Maryland

## Abstract

A 50-year-old male presented to the emergency department with four days of intermittent chest pain and shortness of breath, which progressively worsened in severity. Testing revealed a troponin I greater than 100 times the upper limit of normal and an electrocardiogram with non-specific findings. This case takes the reader through the differential diagnosis and systematic work-up of the deadly causes of chest pain, ultimately leading to this patient’s diagnosis.

**CASE PRESENTATION** (William Fernandez, MD)

A 50-year-old male presented to the emergency department (ED) clutching his chest. He complained of severe generalized chest pain, shortness of breath, left leg pain, and feeling as if he were going to pass out. Over the preceding four days, he had felt a generalized, non-radiating, non-specific chest pain with mild shortness of breath both at rest and with minimal exertion. The discomfort resolved at times spontaneously and at other times with marijuana use. Over these four days the discomfort intermittently recurred, worsening with each occurrence. The morning of his presentation to the ED he woke at 3 am with sudden worsening of the generalized chest discomfort, which at that time had become pressure-like and was associated with an acute worsening of his shortness of breath. Later that morning, he developed severe nausea/vomiting and had a sudden episode of pre-syncope after which he noted an ache-like pain throughout his left leg. At home he tried smoking marijuana to improve his symptoms, but because his symptoms continued to worsen, he presented to the ED for evaluation.

He had a past medical history significant for non-ischemic cardiomyopathy with a left ventricular ejection fraction (LVEF) of 50–55%, remote cerebral vascular accident with residual mild slurred speech, polycystic kidney disease, chronic obstructive pulmonary disease, hypertension, hyperlipidemia, bipolar disorder, and schizoaffective disorder. He did not take any medications and had no known drug allergies. His family history was notable for a myocardial infarction (MI) in his mother at age 56 years, and a cerebral aneurysm rupture in his mother at age 57 years resulting in her death; there was no known paternal history. He related daily tobacco use with a 12.5 pack-year smoking history and daily marijuana use. He denied alcohol or other substance use. He lived in an abandoned apartment with 4–6 roommates, all of whom were active polysubstance abusers.

On arrival to the ED, he was alert and oriented, ill appearing, and in moderate distress due to chest pain. He was afebrile (36.5 degrees Celsius) with a heart rate of 78 beats per minute, blood pressure of 116/69 millimeters of mercury, oxygen saturation of 100% on room air and a respiratory rate of 26 breaths per minute. He weighed 54.4 kilograms (kg) and was 1.65 meters (m) in height with a body mass index of 20.0 kg/m^2^. He was well developed and well nourished. His head was normocephalic and atraumatic with dry mucus membranes. Pupils were equal, round, and reactive to light and accommodation bilaterally with normal extra-occular movements. Sclera were anicteric, and fundi were without papilledema. The neck was supple and without lymphadenopathy or carotid bruits. His lungs were clear to auscultation bilaterally without wheezes, crackles, or rhonchi. He had no retractions but was tachypneic. His heart was regular rate and rhythm without murmurs, rubs, or gallops. The abdomen was soft with normal bowel sounds and without distension, rebound, guarding, or hernias; however, he had tenderness in the epigastric and periumbilical regions. There was no costovertebral angle tenderness. The extremities had no edema, tenderness or deformity. There were 2+ radial and dorsalis pedis pulses bilaterally.

Neurologic examination showed intact cranial nerves II–XII, 5 out of 5 strength throughout all extremities, normal muscle bulk and tone, and intact sensation. His speech was slightly slurred but unchanged from baseline, according to him. His affect and behavior were normal, and he was answering all questions appropriately. He was fully oriented to self, place, and time.

We obtained an electrocardiogram (ECG) (Image 1). Initial laboratory results are shown in Table. Point-of-care ultrasound showed a trace pericardial effusion and a significantly dilated left ventricle with poor systolic function. Computed tomography angiogram (CTA) of the chest, abdomen, and pelvis showed no evidence for acute aortic dissection or injury (Image 2). Cardiomegaly with left ventricular dilation was shown, as well as evidence of advanced atherosclerosis of the pelvic vasculature and polycystic kidneys (previously known). After the CTA of the chest was performed, the patient was taken to the cardiac catheterization laboratory for angiography, which showed an LVEF of 10–20% with akinesis of the inferior wall and hypokinesis of the anterolateral wall of the heart, 2+ mitral regurgitation, severe pulmonary hypertension, and mild diffuse atherosclerotic disease without occlusion of any major vessels. A diagnostic test was then performed, which confirmed the diagnosis.

**CASE DISCUSSION** (Zachary Dezman, MD, MS, MS)

This case illustrates a common challenge for emergency physicians: the simultaneous institution of diagnostics and empiric treatment, while stabilizing an acutely ill patient. I found myself agreeing with every action taken by the treating doctors. The initial history is representative of many patients I see: a middle-aged man with an outsized burden of disease, including polysubstance abuse and psychiatric illness. When I see “chest pain” on a patient’s triage sheet, I think of six “deadly” causes: acute coronary syndrome (ACS), pulmonary embolism (PE), pneumothorax, Boerhaave’s syndrome, aortic dissection, and pericarditis. The patient’s presentation and risk factors could support any of these diagnoses. The challenge is to determine which features of the presentation to focus upon.

The *Journal of the American Medical Association* has endeavored to quantitate physician gestalt through the Rational Clinical Exam series.^1^ For the patient in this case, we see that his prior history of cerebrovascular accident, male sex, and hypertension each slightly raise his risk of ACS.^2^ Tobacco use and a family history of early cardiac disease are classic risk factors for cardiac disease,^3^ but these have variable value for diagnosing ACS in the ED setting.^4^ The patient’s chest pain, including the recent change in the pattern – the exertional component and its association with dyspnea – all increase the probability of ACS. Note that vomiting is associated with MI specifically.^5^ The patient’s ECG is also suggestive of ischemia, with T-wave inversions, and S-T segment depressions. Given these patient features and a pre-test probability of 13% used by Fanaroff et al.,^2^ this patient’s post-test probability of ACS is greater than 80%.^6^ Surveys of physicians have shown that a miss rate of approximately 1% is considered “acceptable” for ACS,^7^ so the physicians in this case are well over the evidentiary threshold needed to pursue a diagnosis ACS!^7^ They should immediately activate the cardiac catheterization lab.

Almost; except for the fact that the treating physicians must have also been concerned about aortic dissection and PE. When a patient complains of pain radiating across anatomical borders, in the way this patient’s chest pain radiated to his leg, I think of vascular disasters such as aortic dissection. Proximal dissections (i.e., type A) are a rare mimic of ACS, and 4% of all proximal dissections are diagnosed while the patient is undergoing cardiac catheterization for presumed acute MI.^8^ Misdiagnosis can be dangerous in these patients as there is a *1–2% cumulative increase in mortality for every hour of delay in treatment*.^8^ Similarly, the patient’s dyspnea, relative hypotension, insidious presentation, and ECG changes could be caused by a pulmonary embolism (PE). The next reasonable step is to rule out these diagnoses and order a CTA of the chest, abdomen, and pelvis. Thankfully, the patient’s imaging did not reveal a dissection or PE.

Once the patient got to the catherization lab, the cardiologist found evidence of heart failure (HF) without an occlusion of the coronaries. The patient’s other laboratory tests had returned by this point, showing an elevated erythrocyte sedimentation rate (ESR), a high C-reactive protein, and a really, *really* high troponin.

A number of non-ischemic conditions can can cause an elevation in troponin. Patients with chronic renal or heart failure, or those with an exacerbation of their obstructive lung disease can have an elevated troponin, but these are usually mild. Strenuous exercise, such as running a marathon or a tachydysrhythmia, can elevate one’s troponin as well, but these too are usually mild. Takostubo or stress cardiomyopathy can present with very high troponins, but the characteristic “apical ballooning,” usually seen on ventriculogram, wasn’t mentioned in the presentation. This leaves myocarditis as the most likely cause of the patient’s presentation.

Myocarditis can have an insidious onset and then present acutely such as an MI with HF.^9^ Troponins in excess of 1,700 nanograms per milliliter (ng/mL) have been recorded in the literature.^10^ ESR and C-reactive protein levels have also been seen in patients with myocarditis. About 7.0% of cases recur, and this patient had undergone a prior nuclear medicine scan in 2002, which could have been related to a prior episode.

This patient’s history shows he is at risk for myocarditis from several causes. He could have had viral myocarditis due to hepatitis B or C, given his substance use history. There are cases of clozapine-induced myocarditis, which the patient might have been exposed to while being treated for his mental illness. Myocarditis has also been associated with lung cancer, and we discovered a new lung mass on this patient’s imaging.

Cardiovascular magnetic resonance (CMR) has become much more common and is now “the primary tool for noninvasive assessment of myocardial inflammation in patients with suspected myocarditis.”^11^ I believe that a CMR was done, which detected edema at the site of injury, seen as high-intensity enhancement on T2-weighted images. Early and late enhancement with gadolinium, which is indicative of irreversible injury, may also have been seen.^11^ These findings would support the final diagnosis of myocarditis.

## CASE OUTCOME

The diagnostic study of choice was CMR. The patient was found to have significant edema throughout the myocardium, with a focus over the anterolateral and inferior walls, seen on T2-weighted images.

While in the cardiac catheterization lab, the patient received an intra-aortic balloon pump (IABP) to support his cardiac perfusion. He was then admitted to the cardiac intensive care unit (CICU) and started empirically on broad-spectrum antibiotics. Serial echocardiograms showed an unchanging LVEF of 10–20% during his first week in the CICU. Further testing did not identify a specific infectious etiology. His LVEF improved to 20–25% on hospital day (HD) 8; the IABP was discontinued, and he was extubated successfully. CMR was then performed, demonstrating the edema described above and making the diagnosis. The remainder of his hospital course was complicated by a moderate retroperitoneal hematoma and dysphagia, both of which were managed medically and improved without further complication. His last echocardiogram before discharge on HD 19 showed an LVEF of 30–35%. He was discharged to a subacute rehabilitation center.

## RESIDENT DISCUSSION

Myocarditis is an inflammatory disease involving the cardiac muscle. It affects 22 per 100,000 persons or around 1.5 million individuals worldwide, annually.^12^ The time course for the disease ranges from acute to chronic, with clinical severity depending on the degree of tissue damage, the underlying etiology, and patient comorbidities.

The most common infectious causes of myocarditis are viral, with over 20 different viruses implicated. Coxsackievirus was the most common up until the 1990s, but more recently parvovirus B-19 and human herpes virus 6 have become more common. Bacteria, fungi, protozoa, and even helminths have been identified as causes of myocarditis. Autoimmune disorders such as systemic lupus erythematosus, giant cell arteritis, and granulomatosis with polyangiitis can cause myocarditis as well. Less common, non-infectious etiologies include various cardiotoxins such as alcohol, cocaine, cyclophosphamide and heavy metals, and hypersensitivity reactions from antibiotics, clozapine, insect bites, and snake bites.^13^

Patients with acute myocarditis will often present with chest pain, dyspnea with or without exertion, unexplained sinus tachycardia, tachypnea, and signs of HF. Physicians evaluating patients with these complaints will often also consider ACS, PE, new-onset HF, or aortic dissection. Items in the patient’s history that are more supportive of myocarditis include a history of a recent mild illness, medication change, illicit drug use, or a lack of cardiovascular risk factors.^13^

Initial testing should include an ECG, chest radiograph (CXR), cardiac biomarkers, and point-of-care cardiac ultrasound if available.^9^ ECG findings can vary dramatically, from sinus tachycardia to diffuse ST-segment elevation with PR-segment depression suggesting pericarditis. The CXR is helpful in identifying cardiomegaly, which is concerning if new, and may show evidence of pulmonary edema. Cardiac biomarkers can be markedly elevated, especially if the disease has involved all four chambers of the heart. Cardiac ultrasound can help confirm the presence of cardiomegaly as well as estimate the LVEF, identify wall motion abnormalities, and evaluate for other possible causes of the patient’s presentation.

CMR can be used to definitively identify myocardial inflammation and make the diagnosis if the patient is clinically stable and the resources are available.^11^ Endomyocardial biopsy is the classic method of diagnosing myocarditis, although it is rarely performed today. The affected tissue may be missed due to sampling error during the biopsy, and myocardial rupture and tamponade are rare but potentially life-threatening complications. Biopsy does have the advantage of being able to provide both a diagnosis and an etiology.

Management of acute myocarditis depends greatly on the patient’s presentation. Severe cases can present with cardiogenic shock secondary to acute HF and may require emergent intubation, ventilatory support and stabilization with the early administration of diuretics. Inotropic support with vasopressors, IABP, or extracorporeal circulatory membrane oxygenation (ECMO) can be used if the patient is hemodynamically unstable with evidence of shock.^14^ Targeted therapies range from appropriate antibiotics for infectious etiologies to intravenous immunoglobulin for autoimmune etiologies. The time-course of therapy depends on the patient’s clinical improvement.^15^ Serial echocardiograms are used both on an inpatient and outpatient basis to determine the patient’s response to therapies.

Prognosis varies with specific etiology and the severity at presentation. Otherwise healthy patients who develop acute myocarditis may return to baseline function if the initial disease process is identified and treated appropriately. Some patients develop chronic myocarditis or have severe enough disease to require cardiac transplantation.^16^

## FINAL DIAGNOSIS

Acute myocarditis due to suspected viral or idiopathic etiology.

## KEY TEACHING POINTS

- A careful history and physical examination are crucial to suspecting mycarditis and initiating the appropriate diagnostics.
- Myocarditis can be acute, subacute, or chronic.
- Initial management is focused on controlling symptoms of HF; hypotensive patients may require inotropes and invasive support with IABP and/or ECMO.
- Cardiac ultrasound is an invaluable tool to assess for the presence and severity of HF.
- CMR is the diagnostic imaging study of choice.

## Figures and Tables

**Image 1:**
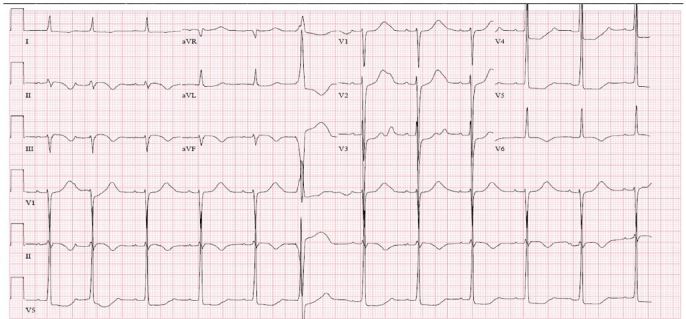
Electrocardiogram done on arrival to the emergency department of a 50-year-old male presenting with chest pain, shortness of breath, leg pain, and pre-syncope.

**Image 2:**
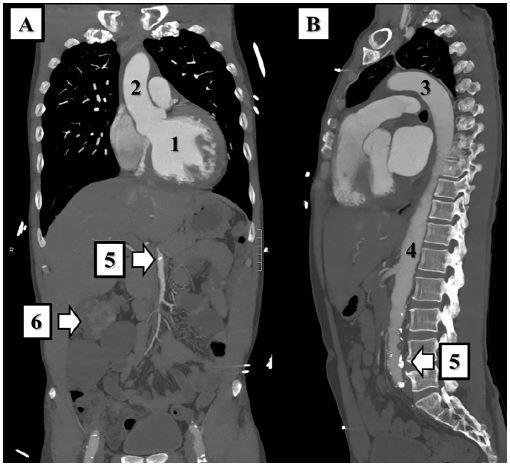
Coronal (A) and sagittal (B) views of the patient’s computed tomography angiogram of the chest, abdomen, and pelvis. The patient’s cardiomegaly can be seen (1), but there is no evidence of dissection at the aortic root (2), arch (3), or descending aorta (4). Calcifications within the abdominal vasculature (5) suggest atherosclerosis. Evidence of the patient’s known polycystic kidney disease was seen (6).

**Table:** Laboratory values of a 50-year-old male presenting with chest pain, shortness of breath, leg pain, and pre-syncope.

		Reference values
Complete blood cell count		
White blood cellsen	20.2 K/mcL	(3.4–9.6 K/mcL)
Hemoglobin	13.2 g/dL	(13.2–16.6 g/dL)
Hematocrit	38.3%	(38.3–48.6%)
Platelets	239 K/mcL	(135–317 K/mcL)
Serum chemistries		
Sodium	139 mmol/L	(136–145 mmol/L)
Potassium	3.6 mmol/L	(3.5–5.0 mmol/L)
Chloride	97 mmol/L	(95–105 mmol/L)
Bicarbonate	29 mmol/L	(22–28 mmol/L)
Blood urea nitrogen	20 mg/dL	(7–18 mg/dL)
Creatinine	1.59 mg/dL	(0.6–1.2 mg/dL)
Magnesium	1.5 mmol/L	(1.5–2.0 mmol/L)
Total protein	7.1 g/dL	(6.0–7.8 g/dL)
Albumin	3.9 g/dL	(3.5–5.5 g/dL)
Total bilirubin	0.7 mg/dL	(0.1–1.0 mg/dL)
Aspartate aminotransferase	1579 u/L	(8–20 u/L)
Alanine aminotransferase	157 u/L	(8–20 u/L)
Alkaline phosphatase	88 u/L	(20–70 u/L)
Additional Labs		
Troponin I	697.0 ng/mL	(<0.034 ng/mL)
Lactate	3.9 mEq/L	(0.3–2.3 mEq/L)
C-reactive protein	6.6 mg/L	(0.0–3.0 mg/L)
Erythrocyte sedimentation rate	55 mm/hr	(0.0–22 mm/hr)

*K/mcL*, thousands per microliter; *mg*, milligrams; *dL*, deciliter; *g*, gram; *mmol*, millimoles; *L*, liter; *u*, units; *ng*, nanogram; *mL*, milliliter; *mEq*, milliequivalents; *mm*, millimeter; *hr*, hour.
